# Structure–function assessment in glaucoma based on perimetric sensitivity and en face optical coherence tomography images of retinal nerve fiber bundles

**DOI:** 10.1038/s41598-023-28917-1

**Published:** 2023-02-13

**Authors:** Muhammed S. Alluwimi, William H. Swanson, Rizwan Malik

**Affiliations:** 1 Department of Optometry, College of Applied Medical Sciences, Qassim University, Qassim, Saudi Arabia; 2 School of Optometry, Indiana University, Bloomington, IN USA; 3 Glaucoma Division, King Khaled Eye Specialist Hospital, Riyadh, Saudi Arabia; 4 Department of Surgery, Sheikh Khalifa Medical City, Abu Dhabi, United Arab Emirates

**Keywords:** Diseases, Health care, Medical research

## Abstract

Many studies have assessed structure–function relations in glaucoma, but most without topographical comparison across the central 30°. We present a method for assessing structure–function relations with en face images of retinal nerve fiber layer (RNFL) bundles allowing topographical comparison across much of this retinal area. Forty-four patients with glaucoma (median age 61 years) were recruited and tested with Optical Coherence Tomography (OCT) and perimetry. Six rectangular volume scans were gathered, and then montaged to provide en face views of the RNFL bundles. We calculated the proportion of locations showing a perimetric defect that also showed an en face RNFL defect; and the proportion of locations falling on an RNFL defect that also showed a perimetric defect. A perimetric defect for a location was defined as a total deviation (TD) value equal to or deeper than -4 dB. We found that the median (IQR) number of locations with abnormal RNFL bundle reflectance that also had abnormal TD was 78% (60%) and for locations with abnormal TD that also had abnormal RNFL bundle reflectance was 75% (44%). We demonstrated a potential approach for structure–function assessment in glaucoma by presenting a topographic reflectance map, confirming results of previous studies and including larger retinal regions.

## Introduction

In the clinical setting, both structural and functional measures are used to diagnose and grade the severity of glaucoma^1,2^. Static automated perimetry (SAP) is commonly used to assess the functional loss in patients with glaucoma^3^. SAP typically utilizes several grids of perimetric locations such as 24-2 and 30-2 grids, in which adjacent perimetric locations are separated by 6° in the vertical and horizontal directions. The use of the 24-2 or 30-2 grids for SAP is associated with sparse sampling of glaucomatous nerve fiber layer (RNFL) defects^4–7^, with discordance between perimetric and structural loss.

Optical Coherence Tomographer (OCT) parameters that have been used for structure–function comparisons include peripapillary RNFL thickness^8,9^, ganglion cell layer thickness^10^ and probability maps of RNFL thickness^11^. En face OCT has the advantage, over peripapillary RNFL thickness, of providing a measure of RNFL integrity directly overlying each retinal test location outside the macula. This method can reveal RNFL loss which is missed by peripapillary RNFL thickness^12^ and not sampled by macular RNFL thickness maps. This method can also decrease the discrepancy that may be found when structure–function models are used to map perimetric locations to optic nerve sectors^13,14^. In addition, en face OCT images can overcome the problem of arcuate-like artifacts in OCT RNFL probability maps in healthy eyes. These artifacts can occur when the distance from the fovea to the region of the greatest RNFL thickness substantially differs from the average distance in healthy individuals^15–17^.

Recently, en face OCT images^18,19^ have allowed assessing the correspondence between SAP damage to the extent of RNFL defect by evaluating the reflectance loss of RNFL bundles at several RNFL depths from the internal limiting membrane (ILM). Therefore, the use of en face OCT image reflectance could be better for determining details of glaucomatous damage than RNFL thickness^20^. As reported in histological studies, RNFL defects may themselves occur at different depths relative to the ILM^21^. In early disease, defects may be confined to deeper depths of the RNFL^22^. Further, RNFL thickness probability maps may miss subtle RNFL loss and en face images may provide one method to overcome this issue^23^.

In a prior study of 10 patients with glaucoma, we used en face OCT images of the RNFL bundles to identify regions of RNFL bundle reflectance loss and compared this with perimetric loss on SAP using the 24-2 grid^24^. To allow spatial mapping of the 48° by 42° sampled by 24-2 grid, a montage of overlapping en face OCT images was used. We demonstrated regions of normal 24-2 SAP sensitivity that exhibited RNF bundles reflectance loss^24^. However, that study was mainly descriptive and limited by a small sample size.

In this study, we aimed to determine the proportion of locations with structural damage (RNFL Bundle reflectance loss) on en face OCT images that did not display perimetric loss.

## Methods

### Participants

We recruited 24 patients from King Khaled Eye Specialist Hospital (KKESH) and 20 patients from Indiana University School of Optometry (IUSO). The age range for the KKESH group was from 34 to 69 years with a median of 56 years, while the age range for the IUSO group was from 48 to 81 years with a median of 69 years. Written informed consent was obtained for each participant. All participants had the opportunity to ask about the purpose and procedures of the study. This study was approved by the King Khaled Eye Specialist Hospital Institutional Review Board and the Indiana University Institutional Review Board. The protocol and procedures for this study adhered to the tenets of the Declaration of Helsinki.

For the KKESH group, consecutive eligible patients who met inclusion and exclusion criteria were recruited from the glaucoma outpatient clinic. For the IUSO group, patients were from a recently published study, which selected them from an ongoing research study based on en face defects that passed through a region near the optic disc^25^.

### Inclusion criteria

All participants underwent a comprehensive eye exam with the best corrected visual acuity of 20/20 except for participants older than 70 years for whom 20/40 visual acuity or better was acceptable. We included patients with an IOP of less than 30 mmHg at enrollment (a prior IOP of 30 mmHg or higher before treatment was acceptable). We included participants who had spherical equivalent between − 6.00 and + 3.00 diopters and cylindrical correction of ≤  ± 3.00 diopters. Additional inclusion criteria were absence of systemic disease affecting visual function, no history of ocular disease except glaucoma in the patient group, absence of ocular surgery within the last six months except uncomplicated cataract surgery or glaucoma surgery, and clear ocular media.

### Exclusion criteria

Exclusion criteria were the presence of ocular disease affecting visual function (except glaucoma) such as diabetic retinopathy, macular degeneration, prior vein occlusion, degenerative myopia, amblyopia, peripheral anterior synechia, medications that affect visual function and advanced cases of epiretinal membrane that prevent the visualization of the RNFL bundles. We also excluded participants who had an abnormal visual field due to neurological disorders such as stroke, postchiasmatic lesion or those who were difficult to image due to poor fixation.

### Equipment

#### Perimetry

The Humphrey Field Analyzer (HFA) was used to measure the perimetric sensitivities. Luminance increment of a circular Goldmann size III (0.43° diameter) white-on-white stimulus was used to measure perimetric sensitivities. We used the 24-2 grid and the Swedish Interactive Threshold Algorithm (SITA). The participants’ spherical equivalent correction was used for perimetric testing.

#### Spectral-domain OCT

Spectralis OCT (Heidelberg Engineering, Heidelberg, Germany) was used to gather images for the central 30° of the retina, that corresponded to much of the area covered by the 24-2 grid. The pupil was dilated with 1% tropicamide, when necessary, to allow rapid OCT scan and to improve the scan quality. The volume files of the vertical dense B-scans, 30 microns apart, were exported from the Spectralis OCT and montaged using a custom Matlab software program (Mathworks Inc., Natwick, MA), details have been previously described^24^.

**Figure 1:**
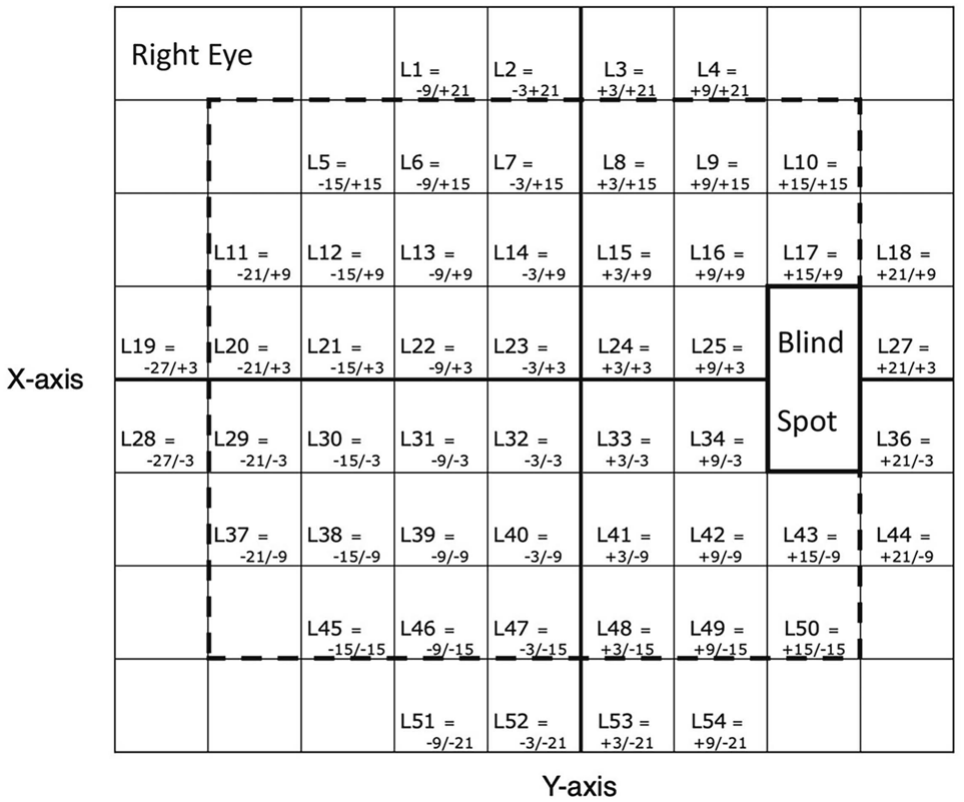
The 54 perimetric locations of the 24-2 grid (after excluding the blind spot) as they are plotted based on x and y axes. Dotted rectangle indicate 38 perimetric locations of the 24-2 grid that were included in the analysis of the current study. This format is for the right eye.

Six overlapping vertical dense scans (rectangles), with high-speed mode, were gathered. These scans were designed to cover adjacent areas of the central 30° of retina^12^. The width and height of the first scan was 15° × 30° and covered the area of the optic disc and adjacent retina. The width and height of second and third scans were both 10° × 20° and covered regions superior and inferior to the macula, respectively. The width and height of the fourth and fifth scans were 20° × 20° and covered temporal superior and inferior regions. The width and height of the sixth scan was 10° × 30° and covered the farthest temporal aspect of the posterior pole. The custom program was used to montage the six volume scans into a single volume scan, that provided en face view at multiple depths from the ILM. This allowed us to visualize RNFL bundle defects at multiple depths from the ILM. We used custom slabs where the depth from the ILM varies with the region of the retina^12^. In some cases, we selected individualized depths to provide the best visualization of the RNFL defect. The 24-2 perimetric locations were superimposed on the montaged en face RNFL image as previously described^24^. The 24-2 locations that corresponded to the en face image protocol are illustrated in Fig. 1.

### Statistical analysis

The number of defective perimetric locations (using 24-2 grid) was compared with presence of RNFL defect on en face OCT. A perimetric defect for a location was defined as any value deeper than − 4.0 dB in the total deviation map. This value was chosen because it is identified as p < 2% or lower at most locations in the 24-2 grid. The RNFL loss was identified based on the low reflectance regions caused by glaucomatous insult, as observed on the en face images of the RNFL bundles^24^. Locations were classified into one of four different categories, to assess concordance between RNFL bundle reflectance loss and perimetric loss, based on the presence of perimetric and retinal nerve fiber bundle defect: (a) en face images of RNFL bundle showed normal RNFL bundles that corresponded with normal perimetric locations; (b) perimetric locations that fell on an RNFL defect and showed perimetric defects; (c) perimetric locations that fell on an RNFL bundle defect *but* did not show a perimetric defect; (d) perimetric locations showed a perimetric defect but the RNFL bundles appeared normal.

The proportion of patients who had glaucomatous defects to RNFL bundles, that were not sampled by perimetry, was reported. For each eye, we calculated the proportion of locations showing a perimetric defect that fell on an RNFL defect; and the proportion of perimetric locations falling on an RNFL defect that also showed perimetric defect. These proportions were calculated based on data derived from total deviation maps.

## Results

**Figure 2:**
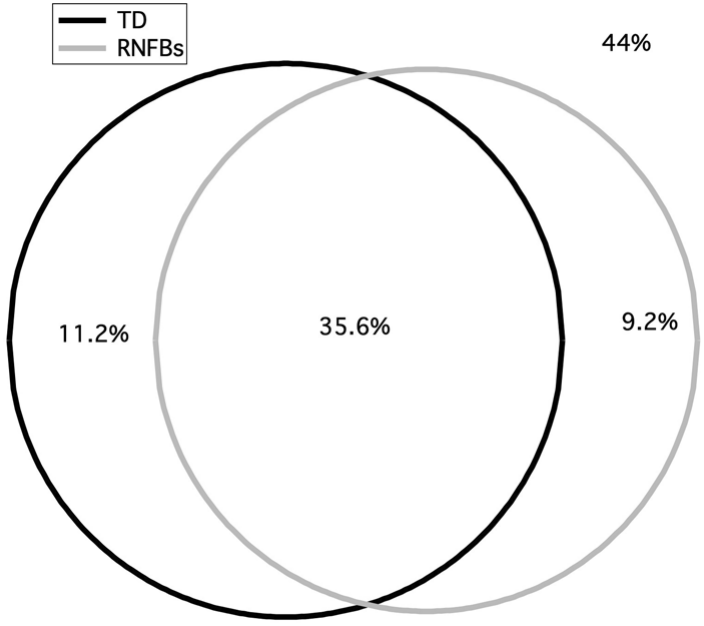
Venn diagram comparing perimetric defect (as derived from the total deviation maps) and the structural defect as observed from the reflectance of the retinal nerve fiber layer bundles (RNFBs) using the optical coherence tomographer (OCT). Each circle represents the proportional area (defective locations) to the number of the locations. The overlapping proportion between the structural and functional defects was 35.6%.

Table 1 shows the demographics of the study population. The mean ± SD age of participants was 61 ± 12 years. The median (IQR) MD across eyes was—4.71 ± 8.25 dB. Out of 44 patients with glaucoma, there were 21 patients who had RNFL defects that were not sampled by the visual field examination when 24-2 grid was used. Figure 2 is a Venn diagram showing the overlap of the proportion of VF locations with abnormal total deviation and low RNFL bundle reflectance. Less than half of all locations, 735 (44%) had normal TD and normal RNFL bundle reflectance; 596 (35.6%) had abnormal TD and abnormal RNFL bundle reflectance, whilst 154 (9.2%) had normal TD with abnormal RNFL bundle reflectance and 187 (11.2%) had abnormal TD with normal RNFL bundle reflectance.

**Table 1:** Characteristics of the patients with glaucoma that we recruited from the King Khaled Eye Specialist Hospital and from the Indiana University School of Optometry.

Variable	Mean ± SD/median [IQR]	Range
Age (years), mean ± SD	60.75 ± 12.33	(34, 83)
SE (D), median [IQR]	− 0.25 [3.93]	(− 7.00, + 5.50)
Axial length (mm), mean ± SD	24.00 ± 1.24	(20.36, 26.29)
MD (dB), median [IQR]	− 4.71 [7.47]	(− 31.3, + 5.90)
PSD, median [IQR]	6.58 [6.11]	(1.53, 14.22)
Intraocular pressure (mmHg), mean ± SD	15 ± 4.3	(6, 26)
Gender (M:F)	28:16	

*SD*  standard deviation, *D* diopter, *IQR* interquartile range, *MD* mean deviation, *PSD* pattern standard deviation, *dB* decibels, *SE* spherical equivalent.

**Figure 3:**
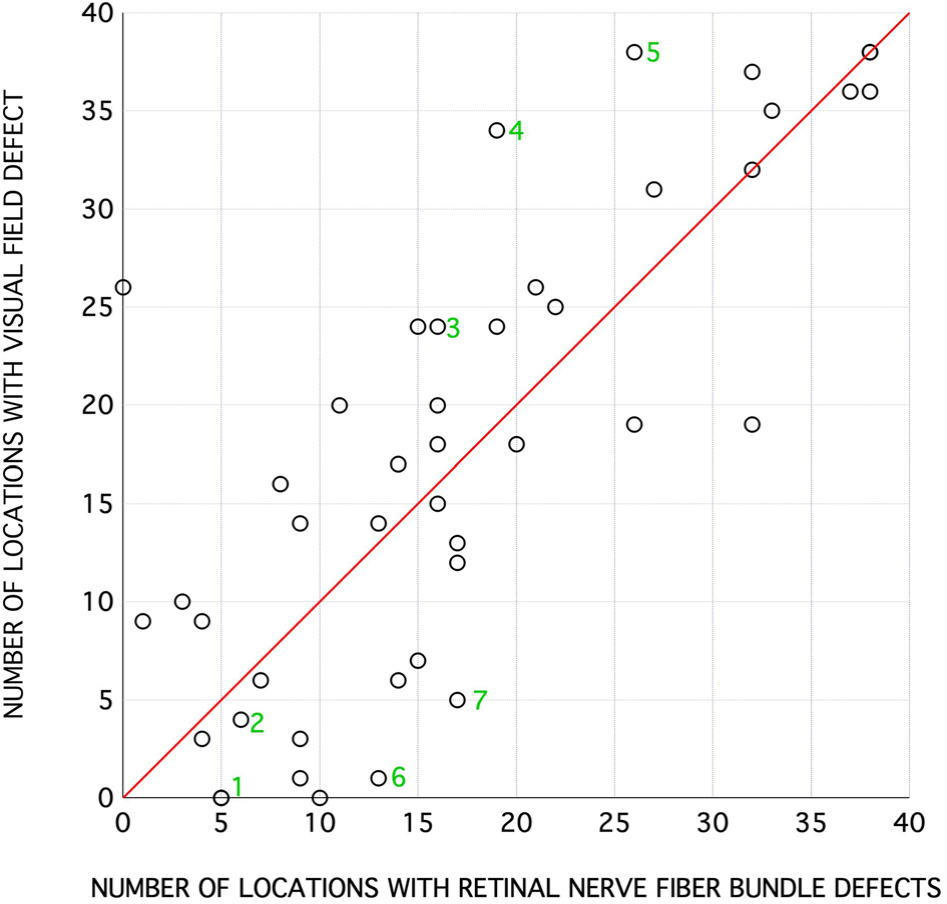
A scatter plot showing the number of defective perimetric locations on y-axis and the number of locations with low reflectance of the RNFL bundles on the x-axis. Symbols with numbers to their right are for the examples shown in Figs. 4 and 5.

**Figure 4:**
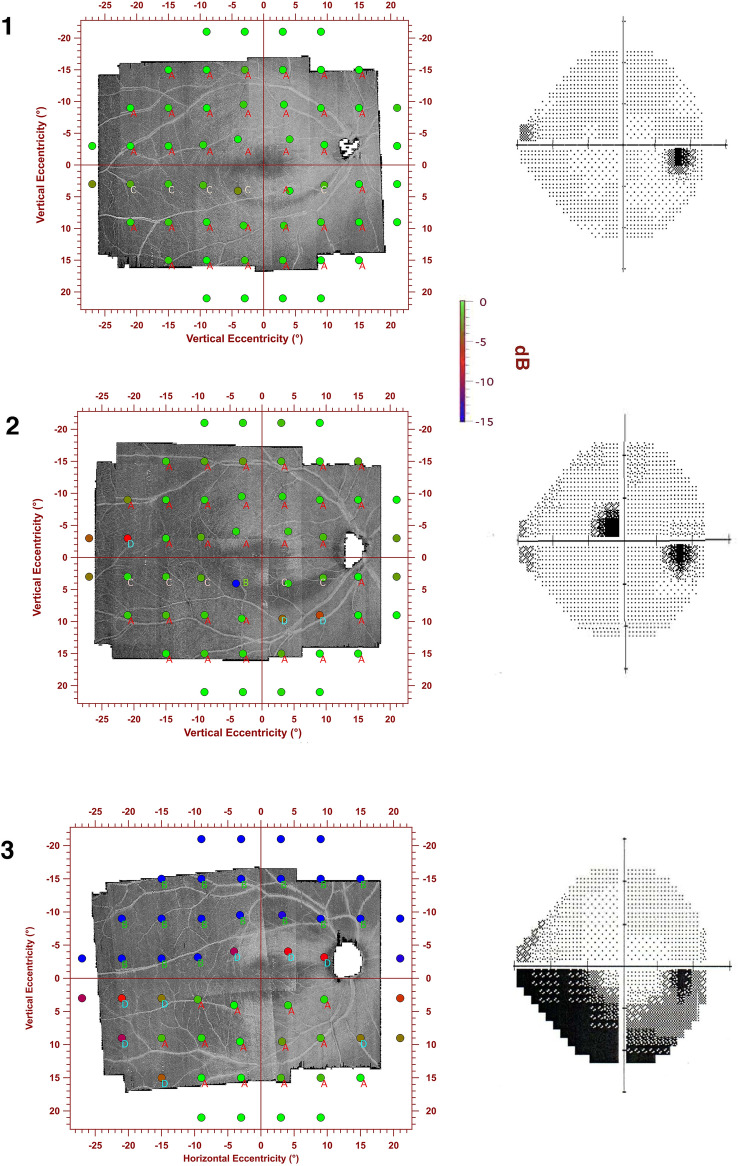
Three eyes from our study, all right eyes. The left column shows perimetric locations overlayed on the en face OCT custom slab image. The perimetric locations are color-coded to indicate the depth of perimetric defect and have been inverted so that the correspondence of perimetric and RNFL bundle reflectance loss can be appreciated. The right column shows the greyscale from the Humphrey Field Analyzer printout. **Case 1**, a 64-year-old with Mean Deviation, MD of − 0.47 dB. **Case 2**, a 70-year-old with MD of − 2.66 dB; **Case 3**, a 54-year-old patient with MD of − 13.64 dB. Some examples of locations with visible RNFL bundle loss and normal perimetric sensitivity (yellow arrows) and locations with abnormal sensitivity and normal RNFL bundle reflectance (cyan arrows) are shown. Letters next to each perimetric location represent one of four categories; (A) is for normal perimetric locations that fell within normal reflectance regions of the RNFL bundles. (B) defective perimetric locations that fell within low reflectance regions of the RNFL bundles. (C) normal perimetric locations that fell within low reflectance regions of the RNFL bundles. (D) defective perimetric locations that fell within normal regions of the RNFL bundles.

**Figure 5:**
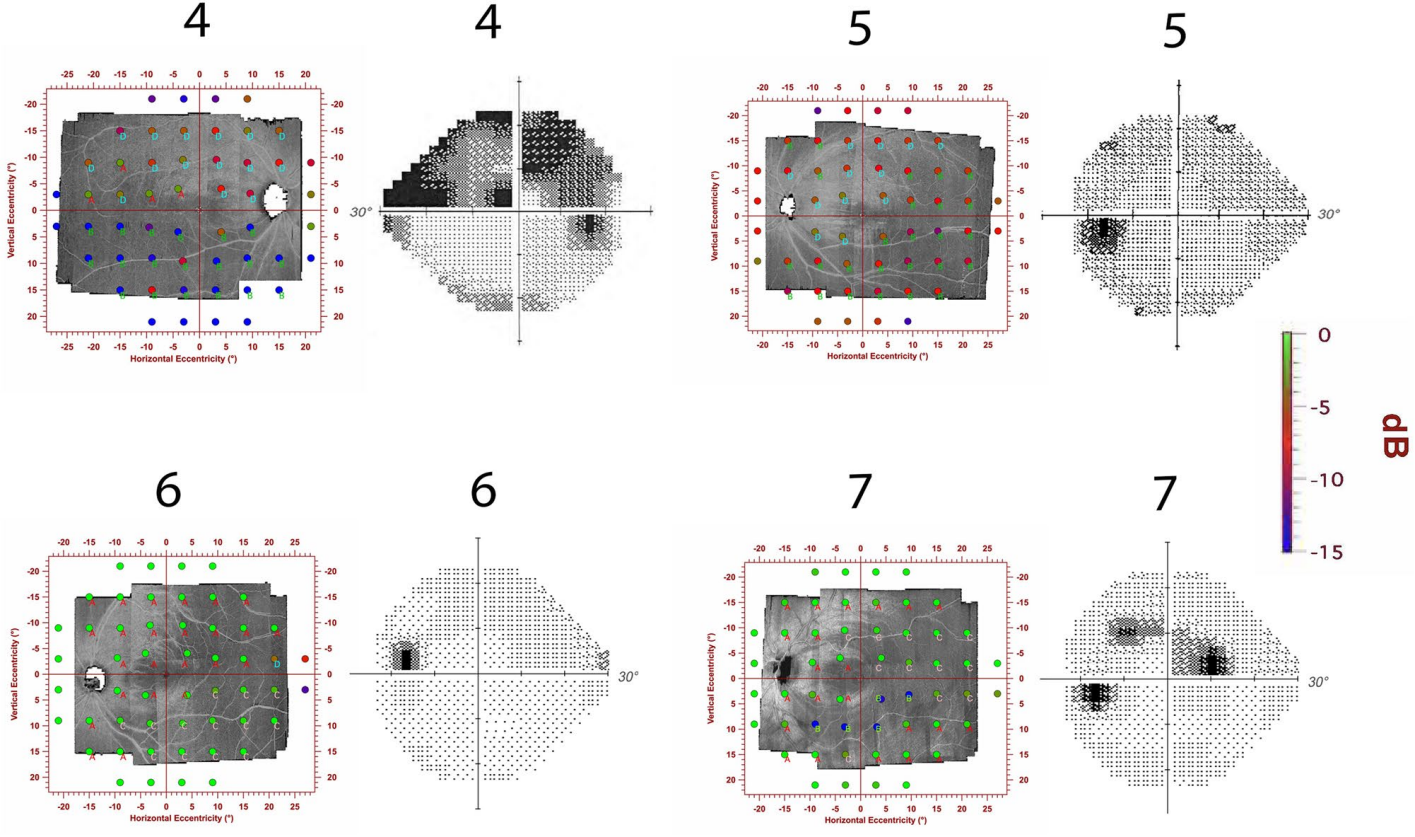
Perimetric locations superimposed on en face images of the RNFL bundles for 4 participants. These 4 participants are extreme cases of structure–function discordance as in Fig. 3. Categories A, B, C and D were described in the Fig. 4 caption. It can be observed that categories "C and D" cover large areas in these examples. The perimetric locations are color-coded to indicate the depth of perimetric defect and have been inverted so that the correspondence of perimetric and RNFL bundle reflectance loss can be appreciated. Next to each en face image is the greyscale from the Humphrey Field Analyzer printout, which is not inverted.

Figure 3 shows data for individuals as the number of defective VF locations that had abnormal RNFL bundle reflectance and vice versa. The median (IQR) was 78% (60%) of locations with abnormal RNFL bundle reflectance with abnormal TD and was 75% (44%) of locations with abnormal TD with abnormal RNFL bundle reflectance. Figure 4 shows eyes of three patients in our study with varying degree of visual field damage (corresponding MDs of − 0.47 dB, − 2.66 dB and − 13.64 dB). The montage shows the en face OCT reflectance image with the overlying 24-2 HFA locations and the corresponding greyscales of the Humphrey VF printout. It can be observed that patient 1 had a very narrow wedge defect that was not sampled by the perimetric testing (category “C”). In patient 2, the perimetric locations were within the RNFL of low reflectance regions (category “B”), but the extent of the RNFL defect did not correspond to the perimetric defect which only showed one defective location. In patient 3, however, most of the defective perimetric locations fell within the RNFL defect. In addition, it can be noted that there are several locations with “D” category where there were defective perimetric locations within the normal RNFL bundle regions. Category “A” indicates normal perimetric locations that fell within a normal reflectance region of RNFL bundles. In Fig. 5, we show extreme cases of structure–function disagreement based on Fig. 3 data.

## Discussion

In this study, we found good agreement between RNFL bundle defects and perimetric loss in patients with glaucoma. Across patients, a median (IQR) of 78% (60%) of locations with abnormal RNFL bundle reflectance had abnormal TD and a median (IQR) of 75% (44%) of locations with abnormal TD also had abnormal RNFL bundle reflectance. RNFL bundles were visualized using en face images, which covered 38 of 52 perimetric locations (after we excluded the two locations around the blind spot). These 38 locations were within ± 15° on the y-axis, and from the locations 21° nasally to 15° temporally. We applied a spatial comparison^24^ to assess the correspondence between RNFL bundle defect and perimetric loss. Despite different methodologies, our findings were consistent with previous studies with regards to the overall, relatively high, magnitude of this agreement between these two measures^11,26^.

In the current study, we used en face images of the RNFL bundles across the central ± 20° of the retina. We then related the RNFL locations with the corresponding perimetric locations of the 24-2 grid. This allowed a direct spatial comparison between the two measures. Prior studies have used other methods to relate individual perimetric sensitivities to OCT measures. Tsamis et al.^11^ used the topographical probability map of the thickness values for ganglion cell plus inner plexiform layer (GCC+), to evaluate the structural and perimetric defects using both 10-2 and 24-2 locations.

Besides the use of the RNFL bundle reflectance in the current study rather than the use of the probability maps, we expanded the evaluation of the structure–function relation to a larger number of locations, 38 locations as compared to 23 in their study. Moreover, it was reported that there is a high between-subject variability observed in the RNFL thickness; this induces arcuate-like defects in the probability maps of the RNFL thickness for the healthy participants^15–17^. This artifact does not occur when the en face images are used, because they are not referenced to normative values. Because the conventional RNFL profile averages the RNFL thickness across the RNFL, subtle defects or defects at certain depths may be missed^27^. However, the use of en face images allows the visualization of the RNFL bundle defects at different depths of the RNFL.

Through this study, we demonstrated the potential of using en face images of the RNFL bundles in determining the characteristics of the glaucomatous damage, with respect to corresponding perimetric defects. En face images enable the visualization of the extent and locations of the structural defects, despite a wide range of glaucomatous defects included in this study. The extent and distribution of structural defects are two critical features to assess the agreement between structural and functional defects in patients with glaucoma, as demonstrated in a prior study from our lab using customized perimetric locations^24^. In the current, study we illustrated the structure–function agreement applying a commonly used grid for testing VF, the 24-2 grid that covers the central ± 20°. Further study with other grids is warranted. For example, we have previously found structure–function agreement between en face images and the 10-2 perimetric grid^28^.

The use of en face images may be limited by variability in the RNFL reflectance among healthy individuals, as previously reported^29,30^. This may result in artefactual glaucomatous defect patterns observed in healthy participants, and may miss glaucomatous defect in patients with glaucoma. Another limitation is that peripheral locations of the 24-2 grid were not covered. The imaging protocol of the current study did not cover 14 out of 52 total locations (excluding the two locations around the blind spot); these locations are beyond 21° temporally, ± 15 in the y-axis, and 15° nasally). These 14 locations are at the edges of the 24-2 grid where these locations might be a source of a lens or lid artifact. On the other hand, the exclusion of these locations may lead to an underestimation of early glaucomatous defects, as the two perimetric locations at the nasal step (temporal raphe of the retina) are excluded. Another limitation is the test–retest variability of the perimetric testing, which may reduce structure–function agreement^31–37^.

In conclusion, we demonstrated a potential approach for structure–function assessment in patients with glaucoma by presenting a topographic map, confirming results of previous studies and including larger retinal regions. We used en face images of the RNFL bundles which covered most of the 24-2 perimetric locations, in order to establish the spatial structure–function comparison. We found a good agreement between structural and functional measures in patients with glaucoma, which was consistent with other studies. Fewer than 10% of locations with abnormal RNFB reflectance were not identified by perimetric loss. More research is warranted to investigate accurate assessment of structure–function relations in early to moderate cases of glaucoma.

## Data Availability

The datasets generated and analysed during the current study will be shared with any research team whose institution executes an approved data use agreement with Indiana University. Interested researchers should contact Indiana University School of Optometry at wilswans@iu.edu, professor William H. Swanson.

## References

[1] Hood DC, Kardon RH (2007). A framework for comparing structural and functional measures of glaucomatous damage. Prog. Retin. Eye Res..

[2] Malik R, Swanson WH, Garway-Heath DF (2012). 'Structure–function relationship' in glaucoma: Past thinking and current concepts. Clin. Exp. Ophthalmol..

[3] Gordon-Bennett PS, Ioannidis AS, Papageorgiou K, Andreou PS (2008). A survey of investigations used for the management of glaucoma in hospital service in the United Kingdom. Eye (Lond.).

[4] Orzalesi N, Miglior S, Lonati C, Rosetti L (1998). Microperimetry of localized retinal nerve fiber layer defects. Vis. Res..

[5] Schiefer U (2001). Evaluation of glaucomatous visual field loss with locally condensed grids using fundus-oriented perimetry (FOP). Eur. J. Ophthalmol..

[6] Westcott MC, Garway-Heath DF, Fitzke FW, Kamal D, Hitchings RA (2002). Use of high spatial resolution perimetry to identify scotomata not apparent with conventional perimetry in the nasal field of glaucomatous subjects. Br. J. Ophthalmol..

[7] Hood DC (2014). A test of a model of glaucomatous damage of the macula with high-density perimetry: Implications for the locations of visual field test points. Transl. Vis. Sci. Technol..

[8] Hood DC, Anderson SC, Wall M, Kardon RH (2007). Structure versus function in glaucoma: An application of a linear model. Invest. Ophthalmol. Vis. Sci..

[9] Harwerth RS, Wheat JL, Fredette MJ, Anderson DR (2010). Linking structure and function in glaucoma. Prog. Retin. Eye Res..

[10] Raza AS (2011). Retinal ganglion cell layer thickness and local visual field sensitivity in glaucoma. Arch. Ophthalmol..

[11] Tsamis E (2020). An automated method for assessing topographical structure–function agreement in abnormal glaucomatous regions. Transl. Vis. Sci. Technol..

[12] Ashimatey BS, King BJ, Burns SA, Swanson WH (2018). Evaluating glaucomatous abnormality in peripapillary optical coherence tomography enface visualisation of the retinal nerve fibre layer reflectance. Ophthalm. Physiol. Opt..

[13] Jansonius NM, Schiefer J, Nevalainen J, Paetzold J, Schiefer U (2012). A mathematical model for describing the retinal nerve fiber bundle trajectories in the human eye: Average course, variability, and influence of refraction, optic disc size and optic disc position. Exp. Eye Res..

[14] Turpin A, McKendrick AM (2021). Improving personalized structure to function mapping from optic nerve head to visual field. Transl. Vis. Sci. Technol..

[15] Leung CK (2010). Retinal nerve fiber layer imaging with spectral-domain optical coherence tomography: Analysis of the retinal nerve fiber layer map for glaucoma detection. Ophthalmology.

[16] Swanson WH, King BJ, Horner DG (2019). Using small samples to evaluate normative reference ranges for retinal imaging measures. Optom. Vis. Sci..

[17] La Bruna S (2022). The OCT RNFL probability map and artifacts resembling glaucomatous damage. Transl. Vis. Sci. Technol..

[18] Vermeer KA, van der Schoot J, Lemij HG, de Boer JF (2012). RPE-normalized RNFL attenuation coefficient maps derived from volumetric OCT imaging for glaucoma assessment. Invest. Ophthalmol. Vis. Sci..

[19] Liu S (2014). Retinal nerve fiber layer reflectance for early glaucoma diagnosis. J. Glaucoma.

[20] Hood DC (2015). Details of glaucomatous damage are better seen on OCT en face images than on OCT retinal nerve fiber layer thickness maps. Invest. Ophthalmol. Vis. Sci..

[21] Ogden TE (1983). Nerve fiber layer of the macaque retina: Retinotopic organization. Invest. Ophthalmol. Vis. Sci..

[22] Radius RL, Anderson DR (1979). The course of axons through the retina and optic nerve head. Arch. Ophthalmol..

[23] Hood DC (2022). Detecting glaucoma with only OCT: Implications for the clinic, research, screening, and AI development. Prog. Retin. Eye Res..

[24] Alluwimi MS, Swanson WH, Malinovsky VE, King BJ (2018). Customizing perimetric locations based on en face images of retinal nerve fiber bundles with glaucomatous damage. Transl. Vis. Sci. Technol..

[25] Swanson WH, King BJ, Burns SA (2021). Interpreting retinal nerve fiber layer reflectance defects based on presence of retinal nerve fiber bundles. Optom. Vis. Sci..

[26] Christopher M (2020). Deep learning approaches predict glaucomatous visual field damage from OCT optic nerve head en face images and retinal nerve fiber layer thickness maps. Ophthalmology.

[27] Cheloni R, Dewsbery SD, Denniss J (2021). A simple subjective evaluation of enface OCT reflectance images distinguishes glaucoma from healthy eyes. Transl. Vis. Sci. Technol..

[28] Alluwimi MS, Swanson WH, Malinovsky VE, King BJ (2018). A basis for customising perimetric locations within the macula in glaucoma. Ophthalm. Physiol. Opt..

[29] Cheloni R, Denniss J (2021). Depth-resolved variations in visibility of retinal nerve fibre bundles across the retina in enface OCT images of healthy eyes. Ophthalm. Physiol. Opt..

[30] Tan O (2021). Focal loss analysis of nerve fiber layer reflectance for glaucoma diagnosis. Transl. Vis. Sci. Technol..

[31] Flammer J, Drance SM, Zulauf M (1984). Differential light threshold. Short- and long-term fluctuation in patients with glaucoma, normal controls, and patients with suspected glaucoma. Arch. Ophthalmol..

[32] Werner EB, Petrig B, Krupin T, Bishop KI (1989). Variability of automated visual fields in clinically stable glaucoma patients. Invest. Ophthalmol. Vis. Sci..

[33] Heijl A, Lindgren A, Lindgren G (1989). Test-retest variability in glaucomatous visual fields. Am. J. Ophthalmol..

[34] Piltz JR, Starita RJ (1990). Test-retest variability in glaucomatous visual fields. Am. J. Ophthalmol..

[35] Henson DB, Chaudry S, Artes PH, Faragher EB, Ansons A (2000). Response variability in the visual field: Comparison of optic neuritis, glaucoma, ocular hypertension, and normal eyes. Invest. Ophthalmol. Vis. Sci..

[36] Artes PH, Iwase A, Ohno Y, Kitazawa Y, Chauhan BC (2002). Properties of perimetric threshold estimates from full threshold, SITA standard, and SITA fast strategies. Invest. Ophthalmol. Vis. Sci..

[37] Russell RA, Malik R, Chauhan BC, Crabb DP, Garway-Heath DF (2012). Improved estimates of visual field progression using bayesian linear regression to integrate structural information in patients with ocular hypertension. Invest. Ophthalmol. Vis. Sci..

